# Complete Genome Sequencing of a Novel Pseudomonas aeruginosa Phage, UF_RH5

**DOI:** 10.1128/mra.00396-23

**Published:** 2023-06-20

**Authors:** Abdolrazagh Hashemi Shahraki, Majid Vahed, Roshan Dinparastisaleh, Mehdi Mirsaeidi

**Affiliations:** a Division of Pulmonary, Critical Care and Sleep, College of Medicine-Jacksonville, University of Florida, Gainesville, Florida, USA; Queens College Department of Biology

## Abstract

Here, we introduce UF_RH5, a novel lytic phage targeting clinically isolated Pseudomonas aeruginosa. It belongs to the *Siphovirus* morphology family, *Septimatrevirus* genus, with a 42,566-bp genome with a GC content of 53.60%, encoding 58 proteins. Under electron microscopy, UF_RH5 exhibits a length of 121 nm and a capsid size of 45 nm.

## ANNOUNCEMENT

The treatment of Pseudomonas aeruginosa infections using antibiotics poses a significant challenge (1). Phage therapy has emerged as a highly effective alternative approach (2). A P. aeruginosa phage named UF_RH5 was isolated by adding filtered wastewater (10 μL) collected from a sewage treatment plant (Alexander Orr Water Treatment Plant located at Miami, FL) to strain DJ06 of P. aeruginosa (400 μL) in logarithmic phase. Using double-layer agar, a single plaque was isolated (3), and the purity of the phage was verified via single plaque isolation. Phage DNA was extracted from phage lysate (5 mL) using a QIAamp MinElute virus kit (Qiagen, USA). The Illumina Nextera XT library preparation kit was used for DNA library preparation, and sequencing was carried out using a Illumina NovaSeq 6000 instrument (paired-end 150-bp cycle). Bcl2fastq v2.20 was utilized to demultiplex reads, and Cutadapt program v2.8 was used to remove sequencing adaptors and low-quality bases (4). Using the read mapper of the STAR package, P. aeruginosa DNA was removed from the data (5). The unmapped paired-end reads were then assembled using MetaWRAP v1.2.00 (6), and the assembled consensus sequences with lengths of >5,000 bp were evaluated by QUAST v5.0.2 (7). Centrifuge v1.04b was utilized to analyze the assembled consensus sequences (8). CheckV v1.01 was applied to evaluate the viral genome completeness and to identify closed genomes (9). The taxonomic identity of the virus was characterized by NCBI BLASTn using the nucleotide collection database (10). Victor was used for phylogenetic analysis (11). GeneMarkS was used to identify open reading frames (ORFs) (12). The genome was annotated based on PHASTER (13) and BLASTp results (10). We used a comprehensive database of nonidentical protein sequences using the default threshold (expect threshold = 0.05 and matrix=blosum62) as the reference database for the BLASTp search. PhageTerm was used to determine the phage termini (14). tRNA sequences were determined by tRNAscan-SE (15). To detect virulence factors and antibiotic resistance factors, ResFinder v4.0 (16) and the Antibiotic Resistance Genes Database (17) were used, respectively. We used default parameters for each software.

**FIG 1:**
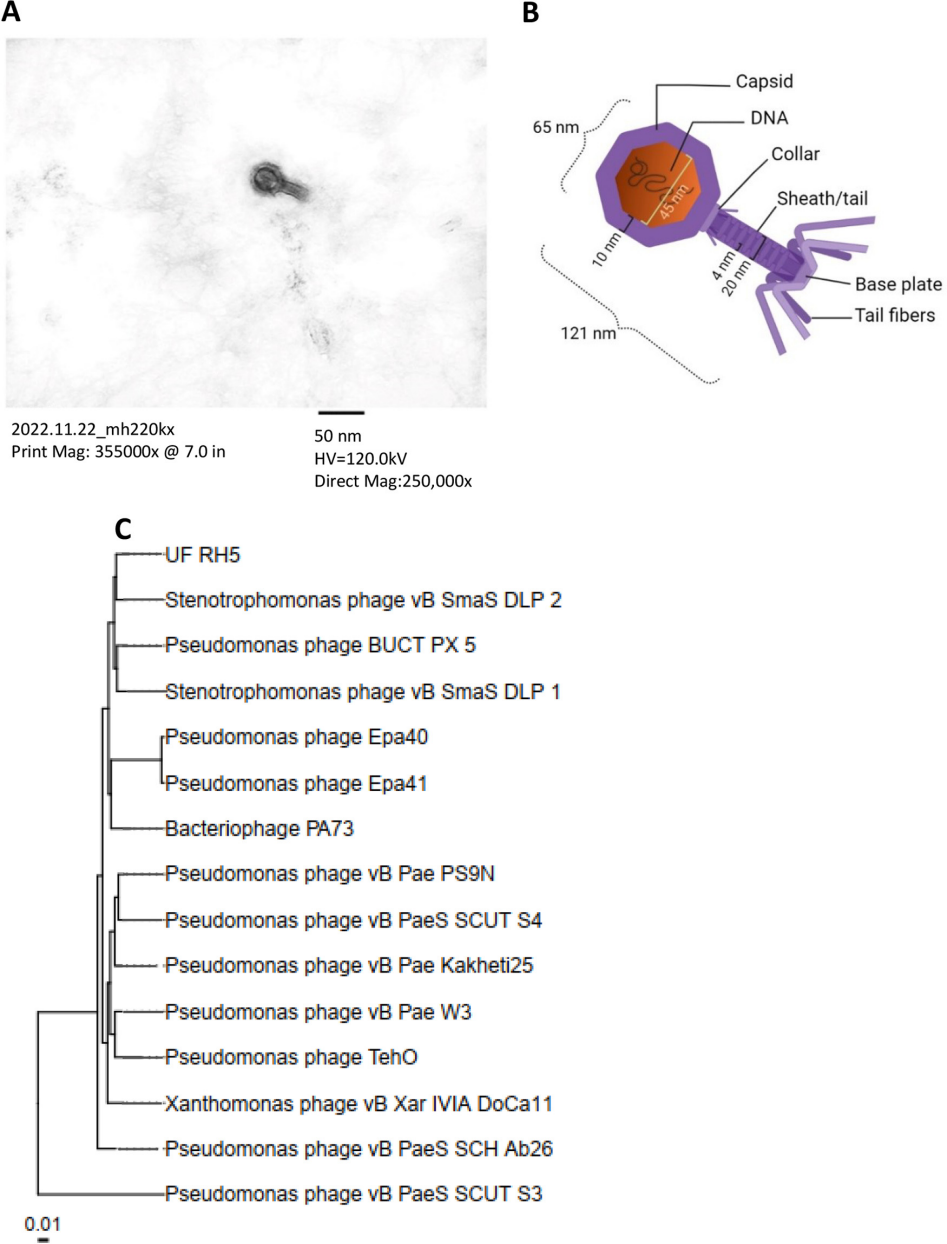
Electron microscopy and phylogenetic analysis of UF_RH5 (18). (A) Negative staining of UF_RH5. High-voltage (HV) is 120 kV with magnification power of 250,000-fold. (B) The schematic image and virus size. (C) The phylogenetic tree illustrates the taxonomic relationship of UF_RH5 with closely related phages belonging to the genus *Septimatrevirus*. The tree was generated using the genome-BLAST distance phylogeny method (GBDP). The scale bar indicates the number of substitutions per site.

The genome of Pseudomonas phage UF_RH5 is a linear double-stranded DNA (dsDNA) with a length of 42,566 bp and a GC content of 53.60%, which was sequenced with an average read coverage of 21,320× and a total of 912,112 reads. PhageTerm predicted a circularly permuted genome for UF_RH5. The genome comprises 58 ORFs and belongs to the *Siphovirus* morphology family and the genus *Septimatrevirus*, as evidenced by sequence similarities with other members (Table 1 and Fig. 1). At genome sequence level, Pseudomonas phage vB_PaeS_SCUT-S4 is close to UF_RH5. The genome of UF_RH5 did not contain tRNA, virulence, or antibiotic-resistant genes. These results confirm the classification of UF_RH5 as a member of the *Siphovirus* morphology family and the genus *Septimatrevirus*.

**TABLE 1:** Genome sequence coverage and nucleotide identity of UF_RH5 with their closest relatives

Phage (GenBank accession no.)^*a*^	Percentage of sequence coverage (similarity) of:							
	UF_RH5	vB Pae-Kakheti25 (JQ307387.1)	vB SmaS-DLP_2 (KR537871.1)	Epa41 (MT118305.1)	Epa40 (MT118304.1)	phipa4 (OK539825.1)	vB PaeS_SCUT-S4 (MK165658.1)	PA73 (DQ163913.1)
UF_RH5 (OQ319036)	100 (100)	95 (96.2)	97 (97.8)	92 (94.8)	91 (94.8)	95 (94.1)	94 (97.2)	94 (94)
vB Pae-Kakheti25 (JQ307387.1)	95 (96.8)	100 (100)	95 (97.1)	91 (96.8)	90 (96.8)	97 (97.9)	98 (96.8)	93 (97.6)
vB SmaS-DLP_2 (KR537871.1)	97 (97.8)	95 (97.08)	100 (100)	89 (97.36)	89 (98.37)	95 (97)	95 (97.3)	93 (98.3)
vB_Pae_PS9N (KM434185.1)	95 (97)	91 (96.8)	89 (97.36)	100 (100)	99 (100)	89 (97.5)	89 (98.34)	92 (97.6)
vB_Xar_IVIA-DoCa11 (ON932085.1)	94 (96.7)	90 (96.8)	89 (98.37)	99 (100)	100 (100)	89 (97.5)	89 (98.34)	92 (97.7)
phipa4 (OK539825.1)	95 (96.9)	97 (97.9)	95 (97)	89 (97.5)	89 (97.5)	100 (100)	98 (96.88)	94 (98)
vB PaeS_SCUT-S4 (MK165658.1)	94 (97.3)	98 (96.8)	95 (97.3)	89 (98.3)	89 (98.3)	98 (96.8)	100 (100)	91 (97.6)
BUCT-PX-5 (OP422637.1)	96 (97.5)	93 (97.6)	93 (98.33)	92 (97.67)	92 (97.75)	94 (98)	91 (97.64)	100 (100)

a All phages are classified as Pseudomonas phage except vB_SmaS-DLP_2 and vB_Xar_IVIA-DoCa11 which is classified as *Stenotrophomonas* and *Xanthomonas* phages, respectively.

### Data availability.

The complete phage genome sequence was deposited in GenBank under the accession number OQ319036. The raw data are available in the NCBI Sequence Read Archive (SRA) under BioProject accession number PRJNA938088, SRA accession number SRR23613576, and BioSample accession number SAMN33424506. Raw sequence data include unmapped nonphage reads.
